# Biological Sexing of a 4000-Year-Old Egyptian Mummy Head to Assess the Potential of Nuclear DNA Recovery from the Most Damaged and Limited Forensic Specimens

**DOI:** 10.3390/genes9030135

**Published:** 2018-03-01

**Authors:** Odile Loreille, Shashikala Ratnayake, Adam L. Bazinet, Timothy B. Stockwell, Daniel D. Sommer, Nadin Rohland, Swapan Mallick, Philip L.F. Johnson, Pontus Skoglund, Anthony J. Onorato, Nicholas H. Bergman, David Reich, Jodi A. Irwin

**Affiliations:** 1 DNA Support Unit, FBI Laboratory, 2501 Investigation Parkway, Quantico, VA 22135, USA; ajonorato@fbi.gov (A.J.O.); jairwin@fbi.gov (J.A.I.); 2 National Biodefense Analysis and Countermeasures Center, 8300 Research Plaza, Fort Detrick, MD 21702, USA; shashikala.ratnayake@nbacc.dhs.gov (S.R.); adam.bazinet@nbacc.dhs.gov (A.L.B.); timothy.stockwell@nbacc.dhs.gov (T.B.S.); daniel.sommer@nbacc.dhs.gov (D.D.S.); Nicholas.Bergman@nbacc.dhs.gov (N.H.B.); 3 Department of Genetics Harvard Medical School, 77 Avenue Louis Pasteur, Boston, MA 02115, USA; nrohland@genetics.med.harvard.edu (N.R.); Swapan_Mallick@hms.harvard.edu (S.M.); reich@genetics.med.harvard.edu (D.R.); 4 Department of Biology, University of Maryland, 1210 Biology-Psychology Building, 4094 Campus Drive, College Park, MD 20742, USA; plfj@umd.edu; 5 The Francis Crick Institute, 1 Midland Rd, London NW1 1AT, UK; pontus.skoglund@gmail.com; 6 Broad Institute of MIT and Harvard, 415 Main Street, Cambridge, MA 02142, USA

**Keywords:** Egypt, ancient DNA, high throughput sequencing, hybridization capture, mitochondrial genome, mtGenome, mummy, sexing

## Abstract

High throughput sequencing (HTS) has been used for a number of years in the field of paleogenomics to facilitate the recovery of small DNA fragments from ancient specimens. Recently, these techniques have also been applied in forensics, where they have been used for the recovery of mitochondrial DNA sequences from samples where traditional PCR-based assays fail because of the very short length of endogenous DNA molecules. Here, we describe the biological sexing of a ~4000-year-old Egyptian mummy using shotgun sequencing and two established methods of biological sex determination (R_X_ and R_Y_), by way of mitochondrial genome analysis as a means of sequence data authentication. This particular case of historical interest increases the potential utility of HTS techniques for forensic purposes by demonstrating that data from the more discriminatory nuclear genome can be recovered from the most damaged specimens, even in cases where mitochondrial DNA cannot be recovered with current PCR-based forensic technologies. Although additional work remains to be done before nuclear DNA recovered via these methods can be used routinely in operational casework for individual identification purposes, these results indicate substantial promise for the retrieval of probative individually identifying DNA data from the most limited and degraded forensic specimens.

## 1. Introduction

On 18 October 2009, the Museum of Fine Arts, Boston (MFA) opened an exhibition called *The Secrets of Tomb 10A: Egypt 2000 BC*. One of the most intriguing items in the collection was a mummified human head discovered over a century ago in the necropolis of Deir el-Bersha (also known as Dayr al-Barshā). The site is located on the east bank of the Nile River in close proximity to the city of Mallawi, approximately 250 km south of Cairo. Deir el-Bersha is known for tombs cut into cliffs of limestone that date back to the Old Kingdom (2686–2181 BC), the First Intermediate Period (about 2100–2040 BC) and the Middle Kingdom (MK; 2040–approx. 1640 BC). During the MK 11th and 12th Dynasties (2040–1783 BC), it served as the chief cemetery for the governors or regional lords (a.k.a. nomarchs) of the 15th Upper Egyptian Nome (a.k.a. the Hare Nome). 

In 1915, Deir el-Bersha was excavated by a joint MFA-Harvard University team directed by George A. Reisner with the assistance of Hanford L. Story and Said Ahmed Said [1]. On 23 April, Reisner’s men began clearing the burial shaft from tomb number 10A. Six days later, at the bottom of a 30-foot pit, they discovered the burial chamber of an early MK governor named Djehutynakht. It is not yet clear whether he is Djehutynakht IV, son of Ahanakht I, or Djehutynakht V, son of Nehri I [1,2,3]. Both were nomarchs of the Hare Nome, and while they shared the same name (which means Thoth [the main local deity] is Strong), there is no evidence they were related. Tomb 10A contained a second occupant: the governor’s wife, who was also called Djehutynakht. Although the tomb had been plundered in antiquity and most of the valuable jewels stolen, many objects were left behind. In fact, the recovered contents of Tomb 10A are considered one of the largest burial assemblages of the MK ever discovered. The funerary equipment includes pottery, canopic jars, models showing men and women in different daily life activities, nearly 60 model boats, and a famous, exquisitely carved and painted processional group composed of a priest and four offering bearers, known as the “Bersha procession” [4]. Befitting their high status, the governor and his wife were both buried in finely decorated rectangular wooden coffins placed within larger coffins, all made of thick cedar of Lebanon boards. Upon discovery, the coffins of the governor were nearly intact with the exception of the head end that had been removed by tomb robbers. The intricate carvings and paintings on the governor’s outer coffin make it an unparalleled masterpiece of MK art (see pictures in [5]). 

**Figure 1:**
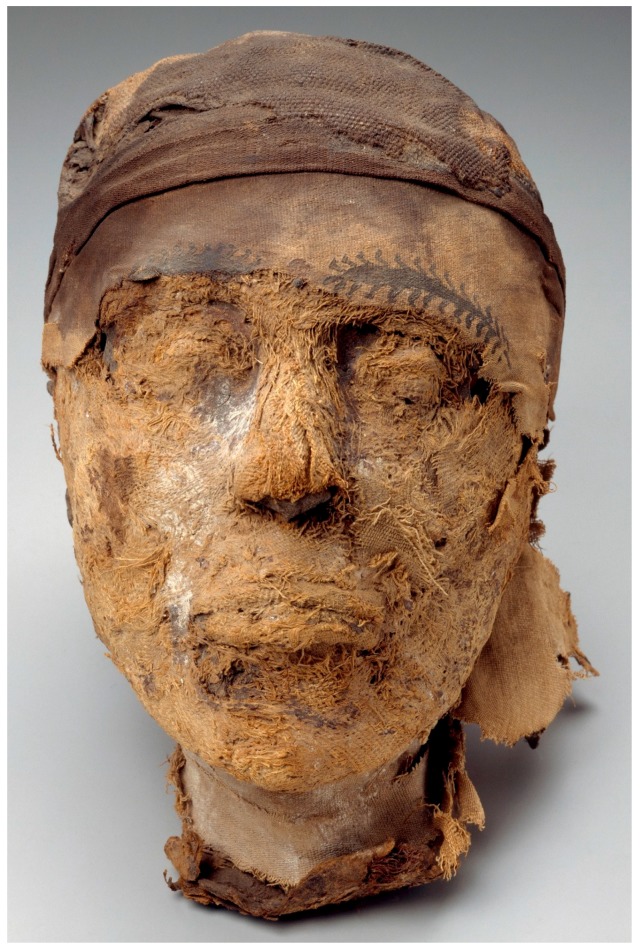
Head of the Djehutynakht mummy (2010–1961 BC). Photograph © 2018 Museum of Fine Arts, Boston, USA.

When the tomb was looted, the mummies of the governor and his wife were damaged by thieves in search of fine jewelry. A torso, originally attributed to Lady Djehutynakht, was found in the far corner of the burial chamber, but was recently argued to belong to the governor after re-excavation of Tomb 10A [6]. A mummified head, which could neither be attributed to the governor nor to his wife, was found atop the governor’s coffin (Figure 1). 

In an effort to learn more, the head was analyzed with computerized tomography (CT) in 2005. The CT scanning of the head revealed extensive bilateral post-mortem alterations of the facial bones [7]. The absence of these bones, together with the lack of comparative data on ancient Egyptian skulls, preclude definitive morphological sex determination; however, the presence of large mastoid processes, robust occipital and temporal regions, and pronounced gonial flaring of the mandible, suggest that the skull more likely belonged to a man [8], (Figure S1). In order to unequivocally determine the biological sex of the individual, the MFA collaborated with the Federal Bureau of Investigation (FBI) Laboratory to perform DNA analysis.

At the time the FBI was contacted, the ancient DNA community had largely given up on the testing of ancient Egyptian human remains. Though DNA extraction and amplification from ancient Egyptian samples had been attempted in the early days of paleogenetics, these initial attempts either resulted in failure (4500-year-old human femurs [9]) or yielded data that turned out to be the product of modern DNA contamination (2400-year-old mummy [10] and 1600-year-old sacred monkey bones from the Saqqara Baboon Galleries [11]), both contaminated with modern human DNA). These early failures prompted studies on DNA survival [9,12]. Together with older research showing that DNA degradation (depurination in particular) is primarily influenced by temperature, pH, oxygen, and water [13], these analyses suggested that Egyptian environmental conditions likely cause DNA to degrade to fragments smaller than 100 base pairs (bp) in just a few hundred years [14,15].

Currently, forensic DNA testing at the FBI, as well as in nearly all global operational laboratories, is based on targeted PCR amplification of fragments ranging from 90–1200 bp in size, followed by size-based capillary electrophoresis for short tandem repeats (STRs) or Sanger sequencing for mitochondrial DNA (mtDNA). These approaches have served the forensic community well over the past twenty years. They present limitations, however, to both the quality and quantity of genetic data that can be recovered from the most challenging specimens. Recently, a number of commercial high throughput sequencing (HTS) assays, designed specifically for forensic applications, have become available [16,17]. While these assays overcome many of the limitations of traditional capillary electrophoresis-based forensic DNA analyses, they are still based on targeted amplification of defined genomic regions. As a result, their utility is limited to samples harboring DNA fragments large enough for PCR amplification.

One of the primary advantages of HTS is that DNA fragments of very short size can be recovered and sequenced, thus obviating the need for targeted PCR. Here, we exploit this feature and describe the use of shotgun sequencing to determine the biological sex of a 4000-year-old Egyptian mummy. 

## 2. Materials and Methods

### 2.1. Tooth Extraction

**Figure 2:**
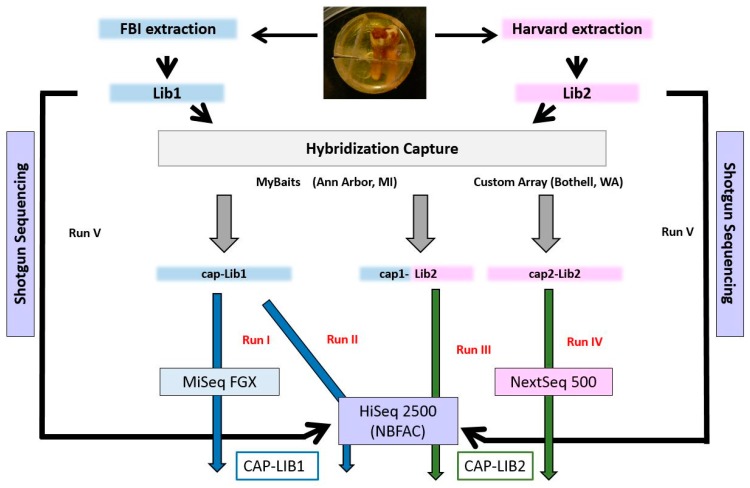
Sequencing strategy in this study. Molecular work performed at the Federal Bureau of Investigation (FBI) Laboratory is in blue, while work performed at Harvard Medical School (HMS) is in pink. Cap1-Lib2 has both colors because the extraction and library preparation of Lib2 was done at the HMS, while the hybridization capture took place at the FBI. CAP-LIB1 is the result of merging data from runs I and II, while CAP-LIB2 is the result of merging runs III and IV. Lib1 and Lib2 were shotgun-sequenced together on the HiSeq at National Bioforensic Analysis Center (NBFAC, run V).

In 2009, a second mandibular molar, in pristine condition, was extracted from the skull using a flexible fiber optic endoscope with grasping forceps [18]. Immediately following its extraction, the molar was placed in a sterile container and sent to a DNA laboratory where it was embedded in epoxy and cut in two parts (see photo in Figure 2). The block containing the crown and upper roots (~2/3 of the tooth) was sent to the FBI Laboratory and later to the Harvard Medical School (HMS) ancient DNA facility.

### 2.2. Strategy for Analyses

Given our understanding that the recovery of DNA from a 4000-year-old Egyptian mummy specimen would be challenging, if not impossible, the first goal was to determine whether any endogenous mitochondrial DNA could be recovered from the sample. On a practical level, the high copy number of the mitochondrial genome (mtGenome) per cell significantly improves the chances of recovering genetic material from ancient samples. Furthermore, because consistently higher HTS coverage is achieved with mtDNA than with nuclear DNA, the authenticity of the genetic data can be more easily addressed. Data authenticity was assessed in three ways. First, the average size of the human mtDNA sequences was determined bioinformatically. Given the ancient Egyptian origin of the sample, the recovered fragments were expected to be very small as a result of DNA degradation over time, while modern contamination would be more likely to contain longer fragments. Second, the occurrence and pattern of one particular type of DNA damage was evaluated. Hydrolytic deamination results in the conversion of cytosine to uracil in the DNA. It is one of the most abundant forms of damage in aged DNA molecules and manifests in the final DNA sequence data as conversion of cytosine to thymine (C-T) or, in the reverse complementary sequence, guanine to adenine (G-A), especially at the ends of molecules [19,20,21]. Finally, the rate of modern DNA contamination in the data was estimated by comparing the data to modern human mtDNA diversity. As mtDNA is a haploid marker, contaminating molecules can be more easily detected than is possible with diploid nuclear DNA.

### 2.3. Sample Preparation

A full description of the sample preparation is provided in the Supplementary Material.

At the FBI Laboratory, DNA was extracted from 105 mg of dentin powder using a silica column-based protocol [22]. In order to remove damaged bases, the extract and associated extraction reagent control (RB) were treated for one hour with the USER^TM^ kit, which contains a mixture of Uracil DNA Glycosylase (UDG) and Endo VIII (New England BioLabs, a.k.a. NEB, Ipswich, MA, USA). At the library preparation stage, a third sample, containing only water, was introduced (negative control or NC). Illumina libraries were generated for each sample (Lib1, Lib1/RB, Lib1/NC) using the NEBNext Ultra II for Illumina kit and NEB looped adaptors. After amplification and indexing, all three libraries were subjected to hybridization capture to enrich for human mtDNA with a MyBait1 kit (Arbor Biosciences, Ann Arbor, MI, USA) and the post-capture products (cap-Lib1; cap-Lib1/RB; cap-Lib1/NC) were sequenced on an Illumina MiSeq FGX at the FBI Laboratory (Figure 2). Having established the presence of endogenous DNA at the FBI Laboratory, the tooth was sent to the HMS, where a second extract and library were prepared. 

At the HMS, DNA was extracted from 68 mg of powder using the protocols published in [23] and [24]. The extract, and associated RB and NC, were treated with UDG according to [25]. By using this treatment, called “partial UDG removal”, almost all the uracil present in the ancient DNA molecules are removed except a few located at the ends of the molecule. Next, three Illumina libraries (Lib2, Lib2/RB, and Lib2/NC) were prepared according to [26]. Fifteen microliters of barcoded Lib2, and 10 µL of Lib2/RB and Lib2/NC were shipped to the FBI Laboratory where 5 μL of each library was dual-indexed with eight cycles of PCR. At the FBI Laboratory, mtDNA hybridization capture was performed on the three Harvard libraries using a MyBait1 kit. Cap1-Lib2/RB and cap1-Lib2/NC were sequenced on the MiSeq FGX while cap-Lib1 and cap1-Lib2 were sequenced together on a HiSeq 2500 at the National Bioforensic Analysis Center (NBFAC). A second mtDNA enrichment on Lib2, Lib2/RB and Lib2/NC was performed at the HMS using a pool of oligonucleotides, synthesized on a microarray (CustomArray Inc., Bothell, WA, USA). Cap2-Lib2, cap2-Lib2/RB, and cap2-Lib2/NC were sequenced at the HMS on a NextSeq 500.

Following confirmation of the mitochondrial DNA data, Lib1 and Lib2 were both shotgun-sequenced on a HiSeq 2500 at NBFAC.

### 2.4. Bioinformatics

Libraries sequenced on the HiSeq or the MiSeq FGX were analyzed at NBFAC. After conversion from BCL to FASTQ format, the HiSeq files were de-multiplexed and the adaptors removed. CutAdapt (v1.9.1; [27]) was used to remove the 7 bp barcodes in Lib2 and cap1-Lib2. For every run except the NextSeq run, paired reads were mapped to the human genome hg19 and the revised Cambridge reference sequence rCRS (NC012910; [28]) using the Burrows–Wheeler Aligner (BWA aln v.0.7.13-r1126; [29]) with the parameters recommended by [30] (“−l 16500 −n 0.01 −o 2”). SAMtools (v1.3.1; [31]) was used to extract mapped reads, merge the BAM files from all HiSeq lanes, and remove mapped reads with a quality score (Q) less than 30. The Picard program v.1.96 [32]) was used to remove duplicates. Data for cap2-Lib2, cap2-lib2/RB and cap2-lib2/NC were analyzed at HMS according to [33]. BAM files of all three alignments were sent to the FBI. At the FBI, all mappings were imported into the CLC Genomics Workbench program (v.10.0.1; Qiagen/CLC bio, Aarhus, Denmark) for visualization. Finally, variants were called using the CLC Fixed Ploidy tool.

#### 2.4.1. Post-Capture Mitochondrial DNA Sequence Analysis

Sequence data produced with cap-Lib1 on two Illumina instruments (runs I and II) were merged into a single CAP-LIB1 file. Similarly, data produced with cap1-Lib2 and cap2-Lib2 (runs III and IV) were merged into a CAP-LIB2 file (Figure 2). For all data sets, duplicates were removed after merging. Reads shorter than or equal to 35 bp were removed from all data sets to avoid spurious microbial alignments (as suggested by [34]) and reads longer than 70 bp (that could potentially originate from modern contaminants) were removed before variant calling. Data observed in the reagent blanks and negative controls were mapped and analyzed using the same parameters.

To assess data authenticity, C-T and G-A errors due to deamination were quantified using MapDamage 2.0 [35]. Modern DNA contamination was estimated using ContamMix [36] and by evaluating the number of human sequences observed in the extraction (RB) and library preparation control (NC) samples. 

The mtDNA haplogroup of the final profile was determined using Phylotree build 17 [37] and HaploGrep 2.0 [38]. Finally, the sequence was compared to other mtDNA sequences from modern and ancient populations stored in GenBank, the European Nucleotide Archives database of the EMBL (ENA) and EMPOP (EDNAP mtDNA population database).

#### 2.4.2. Shotgun Sequence Analysis for Biological Sex Determination

Two established methods that have been successfully applied in a number of previous studies [39,40,41,42,43,44,45,46,47,48,49,50,51,52,53,54,55,56] were used to determine the sex of the individual from whom the tooth was obtained. Biological sex was first determined using R_Y_ [30], which is defined as the number of reads that mapped to chromosome Y (nY) divided by the number of reads mapped to both the X and Y chromosomes (nX + nY). The 95% confidence interval (CI) is defined as R_Y_ ± 1.96 standard error (SE), where SE = √[(R_Y_ x (1 − R_Y_))/nX + nY]. The results are indicative of a female when the upper bound of the 95% CI is less than 0.016, whereas the results are indicative of a male when the lower bound of the CI is greater than 0.075.

The second method, R_X_ [57], compares the number of sequences originating from the X chromosome to the number of sequences originating from the 22 autosomes. Molecular sex is assigned male if the upper bound of the 95% CI is less than 0.60, and female if the lower bound is greater than 0.80 (see R_X_ CI definition in the Supplementary Material).

In order to mitigate against the possible impact of modern contaminants on the calculation, R_X_ and R_Y_ were also determined using only reads showing signs of damage, as these reads are unlikely to have originated from modern contaminants [58]. Read filtering was performed using PMDtools (v0.50; [58]) with a threshold of three, as recommended by the author of the program.

## 3. Results

### 3.1. Data Authentication Based on Captured mtDNA Reads

**Figure 3:**
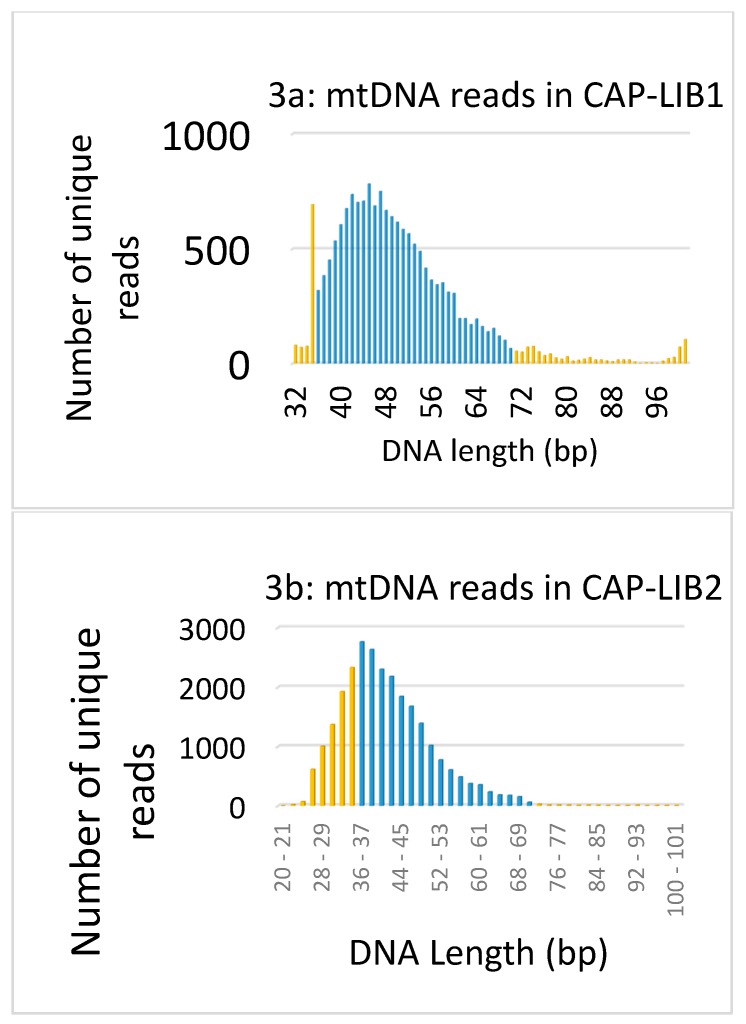
(**a**) Size distribution of the reads that aligned to the mtGenome in CAP-LIB1. Reads ≤35 bp or >70 bp (shown in orange) were removed from the final data to avoid any impact from nonspecific [34] or contaminating reads. The length with the greatest number of reads was 47 bp. (**b**) Size distribution of the reads that aligned to the mtGenome in CAP-LIB2. Only reads >35bp and ≤70 bp (shown in blue) were retained in the final data. The length with the greatest number of reads was 38 bp.

Sequencing results for all four post-capture library runs are presented in Supplementary Table S1. The size distributions of the mapped mtDNA reads from the merged datasets (CAP1-LIB1 and CAP2-LIB2) are presented in Figure 3a,b. The vast majority of reads from the FBI library (CAP1-LIB1) were between 35 and 80 bp, with a mode around 47 bp. The majority of the HMS library reads, on the other hand, were between 25 and 70 bp, with a mode around 38 bp. The shorter average length of the captured mtDNA sequence in the Harvard library (Figure 3b) reflects the efficiency of the extraction protocol from [23] in retaining the smallest molecules. 

**Figure 4:**
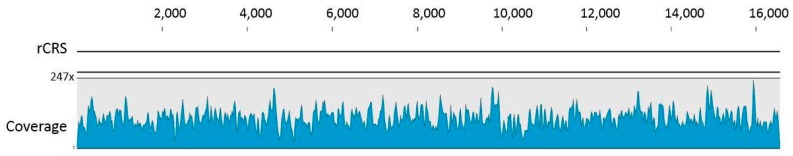
Distribution of 40,910 reads over the entire mtGenome. Sequence coverage at each position ranged from 5× to 247× (average 108×).

The final numbers of unique reads (Q score >30; size ranging from 36 to 70 bp) were 14,408 for CAP-LIB1 and 26,502 for CAP-LIB2 (40,910 reads in total). The total number of mtDNA bases sequenced was ~1.8 million (703,905 for CAP-LIB1 and 1,096,128 for CAP-LIB2). When aligned to the mtGenome, coverage of any given position ranged from 5× to 247×, with an average coverage of 108× (Figure 4). 

### 3.2. Quantification of Deamination

Despite the fact that DNA damage resulting from deamination slowly accumulates over time, deamination does not strictly correlate with sample age. Environmental conditions, in addition to age, can significantly impact DNA damage. High temperatures and humidity generally increase the speed of degradation, while cold and dry climates generally favor preservation. A 22,000-year-old bone preserved in permafrost can exhibit a C-T substitution frequency at the 5’ end of less than 0.20 [59], while a much younger, 6500-year-old human tooth found in Spain can exhibit rates greater than 0.25 [44]. Even if the C-T substitution frequency cannot be used as a strict dating tool, deamination rates are generally expected to be greater than 0.1 in unrepaired DNA extracted from samples greater than 500 years of age that have not been preserved in ice [60]. 

In the present study, the C-T substitution frequency at the 5’ end of the mtDNA reads in Cap-Lib1 was 0.27 (Figure S2A). This high value was somewhat unexpected since the extract had been treated with UDG (an enzyme that cleaves uracil) and only sporadic uracil bases should have remained. These results suggest that the reaction was partially or completely inhibited. Conversely, and as expected for the partial UDG treated cap1-Lib2, very few uracil bases remained in the mtDNA reads, resulting in a nucleotide misincorporation rate of 0.084 at the 5’ end (Figure S2C). Since it has been shown in [25] that a successful “partial UDG treatment” generally results in the misincorporation rate to be reduced by three-fold, it is likely that our USER reaction performed on Lib1 was completely inhibited. 

### 3.3. Contamination

Sequencing statistics for the reagent blank and negative control libraries are presented in Table 1. No signs of measurable human contamination that could have impacted results from the tooth were observed. The vast majority of reads did not map to the human genome and most likely originate from environmental DNA that was present in the tooth, the laboratory, and/or the reagents.

Modern human contamination in the mtDNA data, as assessed using ContamMix, was estimated at 0.6% (95% CI 0.08–1.8%) in CAP-Lib1 and 11.6% in CAP-Lib2 (95% CI 9.7–13.6%). The value for Lib1 is consistent with the rates observed in other ancient DNA studies [44,46,48,49,50,51,52,61,62,63,64,65,66]. Though the estimated contamination rate in Lib2 was somewhat higher, it had no effect on consensus calling for the endogenous molecules, as the same mtGenome profile was recovered from both libraries.

### 3.4. Mitochondrial Haplotype

The mtGenome profile independently obtained from the tooth by the FBI and HMS laboratories were identical and can be found in Table S2. The haplotype (deposited in GenBank under accession number MG736653) belongs to mitochondrial DNA lineage U5b2b5, but the specific sequence has not been previously reported in the 35,942 mtGenomes stored in the NCBI GenBank database (as of October 2017). The sequence closest to the mummy’s belongs to a contemporary individual from Lebanon (KT779192 [67]); however, the two haplotypes still differ at five positions, three of them in the control region (CR). A comparison between the mummy CR and the 26,127 CR sequences from the EMPOP database produced no match. 

To better understand the mtDNA lineage of the mummy in the context of known Egyptian mtDNA diversity, the mummy haplogroup was compared to the mtDNA haplogroup distribution of 668 Egyptians from various modern populations [68,69,70,71,72,73]. The dominant haplogroups among this dataset were haplogroup T (11.98%) and L3 (11.23%; Table S3). Out of the 64 individuals who belonged to haplogroup U, seven belonged to haplogroup U5 (1.05%), and three (0.5%) belonged to one of the U5b subgroups (U5b1c; U5b1d1a; U5b2a5). 

The Djehutynakht sequence was also compared to available ancient human DNA sequences (Table S4). Not surprisingly, no direct matches to the Djehutynakht sequence have been reported. However, related U5b2b sequences have been observed in ancient human remains from Europe, and a haplogroup U5b2c1 haplotype was recently discovered in 2000-year-old remains from Phoenicia [67]. When only the mtDNA sequences recovered from ancient Egyptian human remains are considered, the Djehutynakht sequence most closely resembles a U5a lineage from sample JK2903, a 2000-year-old skeleton from Abusir el-Meleq [74].

### 3.5. Shotgun Sequencing

Shotgun sequencing statistics are presented in Table 2. Although aliquots of libraries Lib1 and Lib2 were mixed (50/50) and sequenced together on the HiSeq, the Lib2 data values were, for all measures, substantially higher than the Lib1 values. This is perhaps due to the smaller overall size of the HMS library fragments (Figure S3). As smaller fragments are known to be preferentially sequenced on Illumina platforms, the smaller average size of the HMS library likely explains the greater numbers of raw paired reads, reads that mapped to the human genome, and unique mapped reads. 

It is also likely that the greater fraction of human DNA recovered from the HMS library (6.57% versus 2.24% in the FBI library) is a direct result of the increased recovery of smaller fragments. 

Due to the extremely degraded state of the endogenous DNA, more of it was likely recovered in the HMS library.

Following the removal of duplicates and reads with low mapping quality scores, Lib1 yielded 1,595,239 reads (1,593,816 nuclear sequences and 1423 mtDNA sequences), while Lib2 yielded 7,691,326 reads (7,687,370 nuclear reads and 3956 mtDNA reads; Table S4). The percentage of mtDNA reads in each of the two libraries was <0.1%, in contrast to the mtDNA capture libraries, where it was 10.9% in Cap-Lib1 and 19.7% in Cap1-Lib2. The average coverage of the mtGenome using shotgun data was too low to produce a full profile. However, when variants could be called (minimum of five reads and a frequency ≥80%), they were in agreement with the profile produced by the hybridization capture data. The average coverage over the entire human genome hg19 was 0.02× for Lib1 and 0.09× for Lib2.

### 3.6. Biological Sex Determination

Both R_Y_ and R_X_ were first calculated using all reads with Q-scores greater than 30 that aligned to the human genome (Table 3; for mapping details, see Table S5). Since the contamination rate in Lib2 was somewhat high (>11%), R_Y_ and R_X_ were also calculated using only reads exhibiting signs of DNA damage (i.e., molecules assumed to be endogenous) (Table 4). For both shotgun libraries (Lib1 and Lib2), the calculated lower bound of the R_Y_ confidence interval was >0.075, regardless of whether all reads or only reads showing signs of deamination were used. Values greater than >0.075 point to a male. Similarly, and again regardless of whether or not undamaged molecules were included in the calculations, data from both Lib1 and Lib2 produced an upper bound for the R_X_ confidence interval that was less than 0.6. For this calculation, values less than 0.6 are consistent with a male assignment.

The DNA length distribution of the shotgun data for Lib1 showed a spike at 35 bp, possibly indicative of spurious microbial alignments (Supplementary Figure S3A; [34]). Therefore, as an extra precaution, R_X_ and R_Y_ were also calculated using only damaged reads >35 bp (467,923 reads). The results were as follows: R_X_ = 0.4369 (95% CI 0.4134–**0.4603**) and R_Y_ = 0.0879 (95% CI **0.0827**–0.093). The male sex assignment remained the same.

## 4. Discussion

### 4.1. Origins of the Mummy

For nearly a century, the contents of Tomb 10A have been studied by archaeologists, philologists, and art historians. Based on the styles of pottery, the many wooden sculptures and, of course, the decorations and texts on governor Djehutynakht’s coffins, the tomb has been firmly dated to within a generation between the end of the 11th and the beginning of the 12th Dynasties (1961–2010 BC; [3,75]). The head is clearly original to the tomb. While, for genetic testing purposes, carbon dating of the specimen would have been ideal to further establish its age, additional manipulations were unfortunately impossible, given the value of the head as an object of art. However, several features of both the head and the tomb point clearly to the head originating from one of the original tomb occupants. For one, there is no evidence that the tomb was later reused despite it having been looted in antiquity. No artifacts or tomb contents dating to a later period have been found; in fact, additional human bones found in one of the shafts of Tomb 10A during a more recent 2009 expedition at Deir el-Bersha still support the conclusion that the chamber was used as the resting place for only two individuals [6]. The preparation of the head itself is consistent with the dating of the artifacts in the tomb (1961–2010 BC). The head displays rather archaic mummification practices, namely, facial features that were modeled in linen and then painted. This type of treatment was common during the Old Kingdom, but progressively disappeared during the MK [76,77,78]. Indeed, the eyes and lips of Djehutynakht’s head were padded and his eyebrows painted (Figure 1). Similar features have been observed in another Deir-el Bersha mummy slightly predating Djehutynakht’s era. The mummy of Henu, recovered from a completely undisturbed late First Intermediate Period (~2100–2040 BC) tomb, exhibits the same characteristics regarding facial modeling [79]. As these particular mummification practices did not persist through the MK and later, this feature supports the position that the Tomb 10A head is contemporaneous with the rest of the tomb’s early MK contents [76]. All told, from art history, anthropological, and archaeological perspectives, the facts point to the mummy head belonging to one of the original Tomb 10A occupants: a MK nomarch of the late 11th or early 12th Dynasty or his wife.

### 4.2. Eurasian mtDNA Haplogroups in Ancient Egyptians

At the time DNA testing was performed on the tooth, and for reasons previously discussed, very little had been published on DNA recovery from ancient Egyptian human remains. Only one publication including HTS and quality control measures was available in early 2016, which described the mtGenome sequencing of an Egyptian mummy from the Greco-Roman period. The individual belonged to mtDNA haplogroup I2 [80]. Two other studies describing mtDNA recovery from ancient African samples were also available at the time, but centered on skeletons from more southern regions of the continent. One described the L0d2c1c lineage mtGenome of a 2330-year-old male skeleton from South Africa [81], while the other described the recovery of a L3x2a mtGenome from the remains of a 4500-year-old individual from Ethiopia [82]. 

Given limited available data and the fact that U5 is the dominant mitochondrial haplogroup found among hunter-gatherers in Europe [83,84], the recovery of a haplogroup U5b2b5 sequence from the mummy of Djehutynakht raises the question of data authenticity, despite the molecular metrics suggesting otherwise. When the mummy’s mtDNA sequence is viewed in the context of modern mtDNA diversity, however, the observed U5 lineage could potentially reflect interactions between Egypt and the Near East that date as far back as the Predynastic and Early Dynastic periods [85]. Trade between Egypt and the Near East is evidenced by, among other things, ceramic imports to Egypt [86]. In addition, dwellings similar to those found in Palestine suggest some immigration to Egypt from more arid Near Eastern areas from the late Predynastic to the Old Kingdom [85,87]. Both trade and immigration between Egypt and the Near East continued to increase over time. Demand in Egypt for cedar of Lebanon wood (a wood available and harvested in Lebanon and Syria during the MK) led to the further establishment of trade routes between Egypt and the Levant [85,86]. It is interesting, and perhaps not coincidental, that the individual with the mtDNA sequence most similar to Djehutynakht comes from a Lebanese individual.

On top of this historical information offering an explanation for the observed mtDNA data are now additional, recently published, mtGenomes from Africa, and Egypt in particular. MtDNA haplotypes recently obtained from ancient human remains from sub-Saharan Africa belong only to haplogroup L subgroups [65,88]. However, nearly all of the remains excavated in the Northern part of the continent belong to Eurasian mtDNA lineages [63,67,74,89,90]. In fact, of the 114 mtDNA genomes now available from northern African ancient human remains, only one belongs to an African lineage (L3 observed in a skeleton from Abusir el-Meleq [74]). The deep presence of Eurasian mtDNA lineages in Northern Africa has, therefore, been clearly established with these recent reports and offers further support for the authenticity of the Eurasian mtDNA sequence observed in the Djehutynakht mummy. In the present study, Near Eastern influence has been found in an individual of high social status who lived in Upper Egypt during the Middle Kingdom. 

### 4.3. Perspectives for Forensic Laboratories

All currently employed capillary electrophoresis-based human DNA identification methods, as well as recently released commercial HTS assays developed for forensic applications, are based on targeted PCR approaches requiring endogenous DNA fragments, sometimes as small as 70 bp [91] but generally >100 bp. In forensic cases involving the most limited and degraded specimens (e.g., burnt bones, touch DNA, or single hair shafts), the DNA is often too damaged to yield amplicons of that size. Even in those instances for which DNA typing is successful with currently employed technologies, results are generally purposely limited to small regions of the mitochondrial DNA control region. Previous DNA analyses of the Djehutynakht tooth, prior to its receipt by the FBI, are consistent with these trends. Testing performed on the root end of the tooth (Figure 2) between 2009 and 2012 by two different laboratories that used traditional PCR approaches and amplicons as small as 80 bp failed. 

The utility of capture-based and shotgun HTS approaches for these types of difficult samples has been recognized for some time in forensics. A number of studies have demonstrated the utility of HTS in recovering DNA from samples recalcitrant to standard PCR. DNA has been recovered from severely degraded human remains [92,93,94,95] as well as single shed hairs [96]. All work to date, however, has focused exclusively on the mitochondrial genome. The mtDNA results produced here further support the use of HTS techniques for the recovery of accurate and reliable complete mtGenome data from severely degraded specimens, and from capture-based data in particular. However, the data described here also expand on these mtDNA-only approaches by demonstrating the recovery of authentic data from the much more discriminating nuclear genome. 

Nuclear DNA testing in forensics is currently based on autosomal short tandem repeat (STR) loci, and, thus, STRs would be the logical target marker for individual identification purposes. Unfortunately, due to their relatively large size, successful targeted amplification of STRs is rare with highly degraded samples. In addition, STRs are difficult to recover from shotgun data in these types of specimens. Not only is the full length of the repeat region rarely intact, but small recovered fragments of repetitive sequence are also unlikely to include enough genome-specific flanking regions to allow for proper mapping. Single nucleotide polymorphisms (SNPs), however, can be used for individual identification [97,98,99], and these could be typed using hybridization capture and HTS. SNPs are already being targeted by the ancient DNA community to determine Y haplogroups [74,88,100], assess kinship [89], and test models of human population history and evolution [41,61,62,64,65,66,100,101,102]. For these types of questions, thousands of nuclear SNPs are often targeted, and low coverage of even a subset is generally sufficient to answer the question at hand.

For forensics and other regulated disciplines (e.g., clinical genetics), where questions pertain specifically to individual genetic profiles, strict metrics for profile accuracy and reliability must be met. For example, adequate depth of coverage must be established to ensure SNP genotyping accuracy, and proper analytical thresholds must be set to capture stochasticity in allelic sampling, laboratory processes, and data analysis workflows [103,104]. These data quality metrics must furthermore be met with time-efficient and cost-effective workflows. Thus, while proof of principle studies in the ancient DNA and, now, forensics disciplines demonstrate recovery of endogenous nuclear DNA from severely compromised specimens, more developmental work is clearly required to enable standard forensic application of these techniques.

## 5. Conclusions

Over the past year, molecular techniques developed and routinely used by the ancient DNA community have finally permitted the recovery of endogenous DNA from ancient Egyptian remains. Here, those techniques were employed to recover the complete mtGenome of the 4000-year-old mummy, Djehutynakht, and to determine that the biological sex was male. Although these approaches have been recently adopted and implemented by the forensic community to develop probative mitochondrial DNA data from the most degraded specimens, further work is needed for the recovery of individually identifiable nuclear DNA markers. Assays will need to be optimized and cost-effective workflows developed to achieve accurate and reliable calls from limited quantities of damaged nuclear DNA. In the meantime, the work described here represents a first step towards further improving forensic DNA testing capabilities in such cases.

## Figures and Tables

**Table 1:** Sequencing statistics for the extraction reagent control (RB) and high throughput sequencing (HTS) reagent negative controls (NC).

		Total # Reads	# Unique Reads Mapped to hg19	# Unique Reads Mapped to the mtGenome
Cap-Lib1	RB	428,192	560	0
	NC	47,414	121	0
Cap2-Lib2	RB	744,228	570	10
	NC	250,282	128	7

**Table 2:** Shotgun sequencing mapping statistics.

	Sequence Statistics	FBI Shotgun-Lib1	HMS Shotgun-Lib2
a	Number of raw paired reads	164,451,485	266,162,607
b	Number of reads mapped to the human genome hg19 and rCRS	3,692,504	19,485,309
c	Percentage of endogenous human DNA	2.24%	6.57%
d	Number of unique human reads with Q >30	1,595,239	7,691,326
e	Average coverage hg19 Average coverage mtGenome	0.02× 4.21×	0.09× 8.93×
f	Number of unique mapped human reads with signs of damage (PMDtools score >3)	518,381	344,995

**Table 3:** R_X_ and R_Y_ determination using unique mapped reads with Q > 30.

Samples	Lib1	Sex	Lib2	Sex
Mapped reads	1,595,239		7,691,326	
Mapped to X	37,605		176,181	
Mapped to Y	3732		16,469	
R_Y_	0.090		0.0855	
95% CI	**0.087**–0.093	♂	**0.084**–0.087	♂
R_X_	0.45		0.433	
95% CI	0.429–**0.471**	♂	0.4–**0.466**	♂

Bold symbolizes the CI used to determine sex.

**Table 4:** R_X_ and R_Y_ determination using unique mapped reads that showed signs of DNA damage.

Samples	Lib1	Sex	Lib2	Sex
Mapped reads	518,381		344,995	
Mapped to X	11,512		6688	
Mapped to Y	1133		637	
R_Y_	0.0896		0.087	
95% CI	**0.0846**–0.0946	♂	**0.0805**–0.0934	♂
R_X_	0.4216		0.3638	
95% CI	0.3987–**0.4447**	♂	0.3261–**0.4015**	♂

Bold symbolizes the CI used to determine sex.

## Supplementary Materials

The following are available online at http://www.mdpi.com/2073-4425/9/3/135/s1. Figure S1: 3D views of the mummy’s skull; Figure S2: MapDamage analyses; Figure S3: DNA length distribution in shotgun sequencing data. Table S1: Sequencing statistics for post capture libraries; Table S2: Mitochondrial DNA profile of the Djehutynakht mummy; Table S3: Haplogroup distribution among 668 modern Egyptians; Table S4: U5b2b individuals in ancient populations; Table S5: Mapped read distributions used to determine R_X_ and R_Y_.
