# Supplementary material for: Biological Sexing of a 4000-Year-Old Egyptian Mummy Head to Assess the Potential of Nuclear DNA Recovery from the Most Damaged and Limited Forensic Specimens
Source: Genes (Basel). 2018 Mar 1;9(3):135. doi: 10.3390/genes9030135 (PMC5867856; doi:10.3390/genes9030135)
Supplement: Supplementary file 1 [file genes-09-00135-s001.zip › Sup files/Sup Figures.pdf]

Supplementary Figures

Figure S1. 3D surface-rendered views of the skull obtained with a flat panel volume CT scanner prototype from Siemens Medical Solutions. The arrow shows the tooth that was extracted for DNA analysis (courtesy of Dr. R. Gupta).

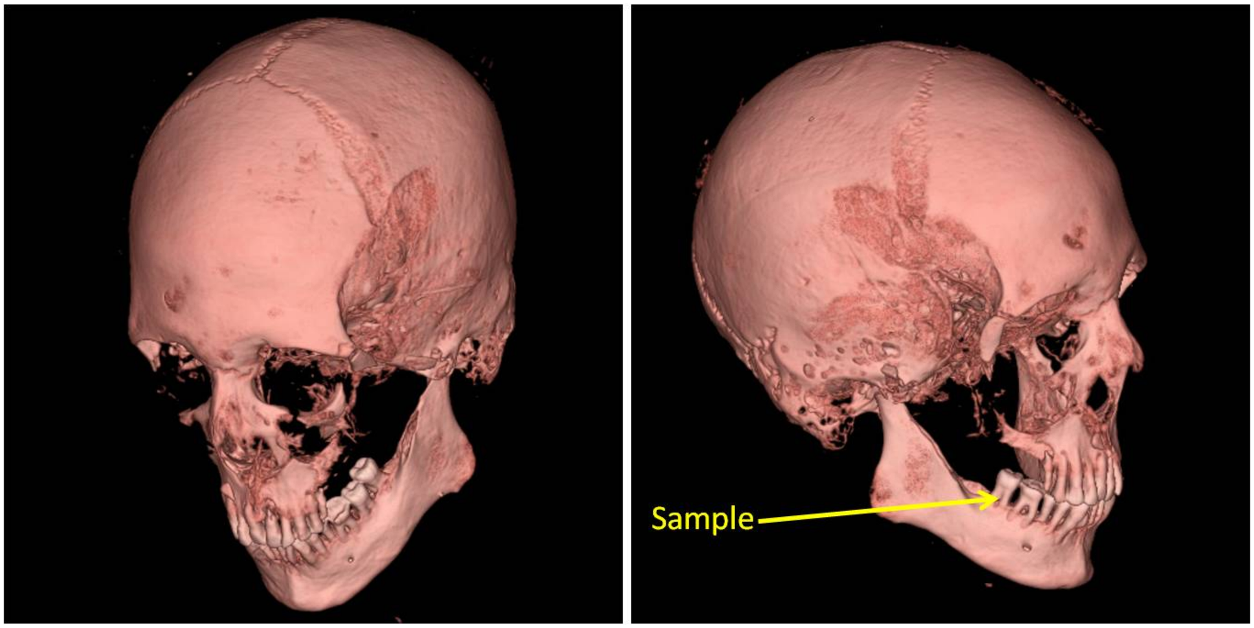

Figure S2. MapDamage analyses for three datasets. Within each panel, the top four subpanels show nucleotide base composition in the reference sequence +/- 10 base pairs of the fragmentation breakpoints. The striking base composition immediately outside the sequenced fragment: position -1 at 5’ end (see orange arrows) and position +1 at the 3’ end, is a hallmark of ancient DNA and arises as a result of the biochemical processes involved in ancient DNA fragmentation [1]. The bottom subpanels show misincorporation rates when comparing the reads to the reference sequence, with C→ T changes in red , G→ A changes in blue, and all other misincorporations in gray.

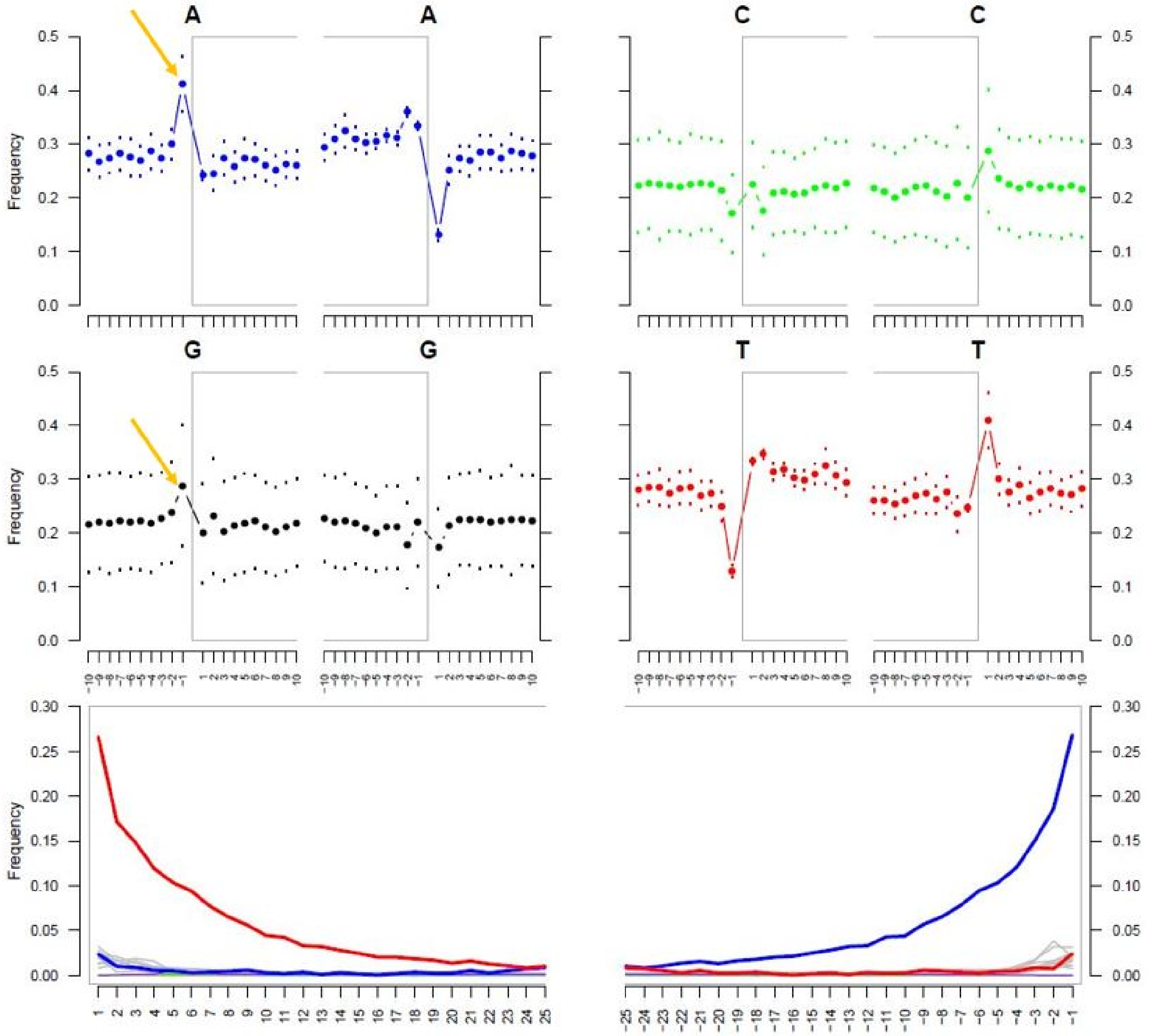

A: Cap-Lib1. mtDNA capture data for the FBI Library (18,691 reads). In the bottom subpanel, deamination rates (C→T at 5’ end; complement at 3’ end) decrease toward the middle of the fragment, as expected in ancient DNA sequence [1].

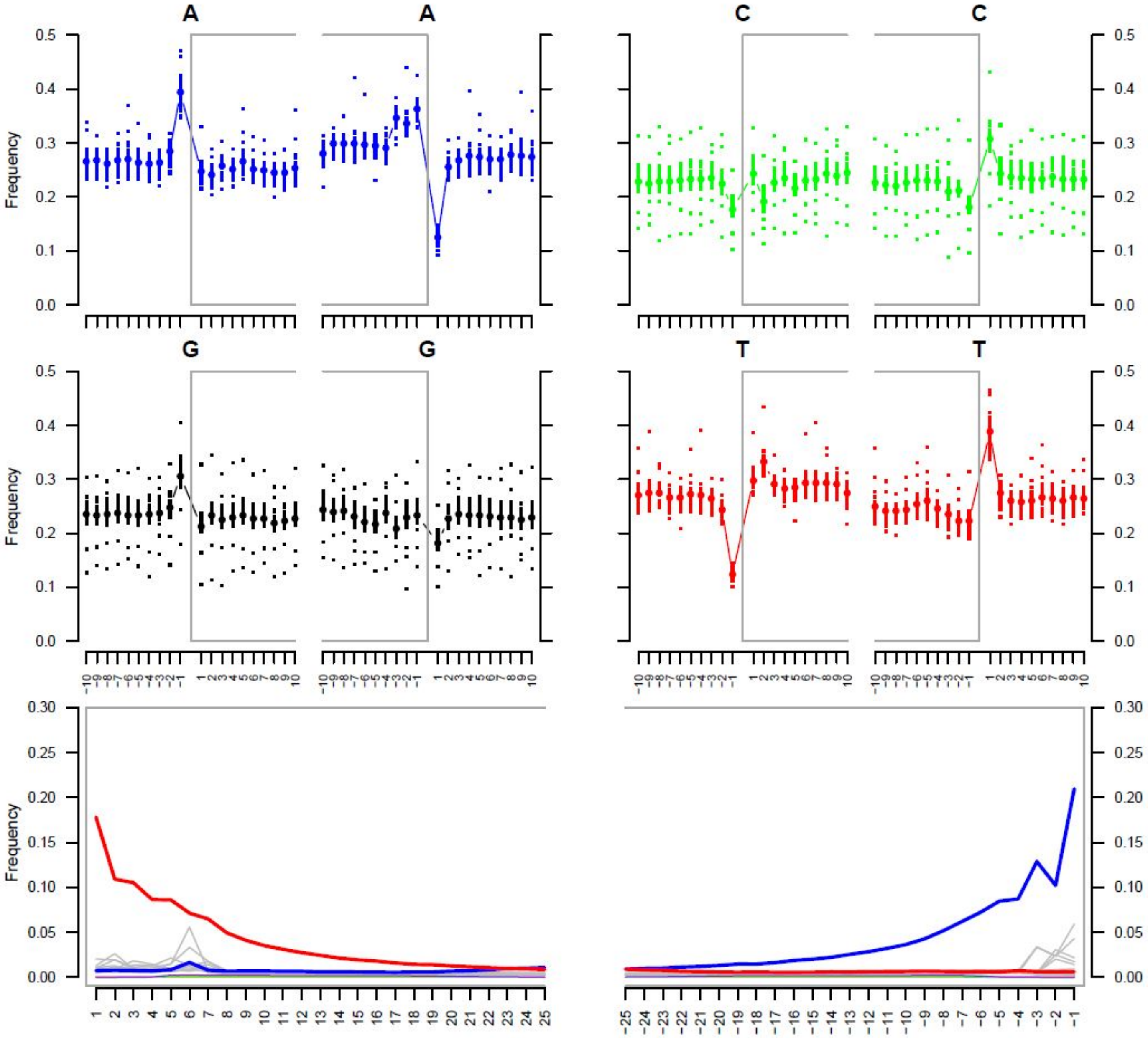

B: Lib1-Shotgun sequencing data (1,595,239 reads).

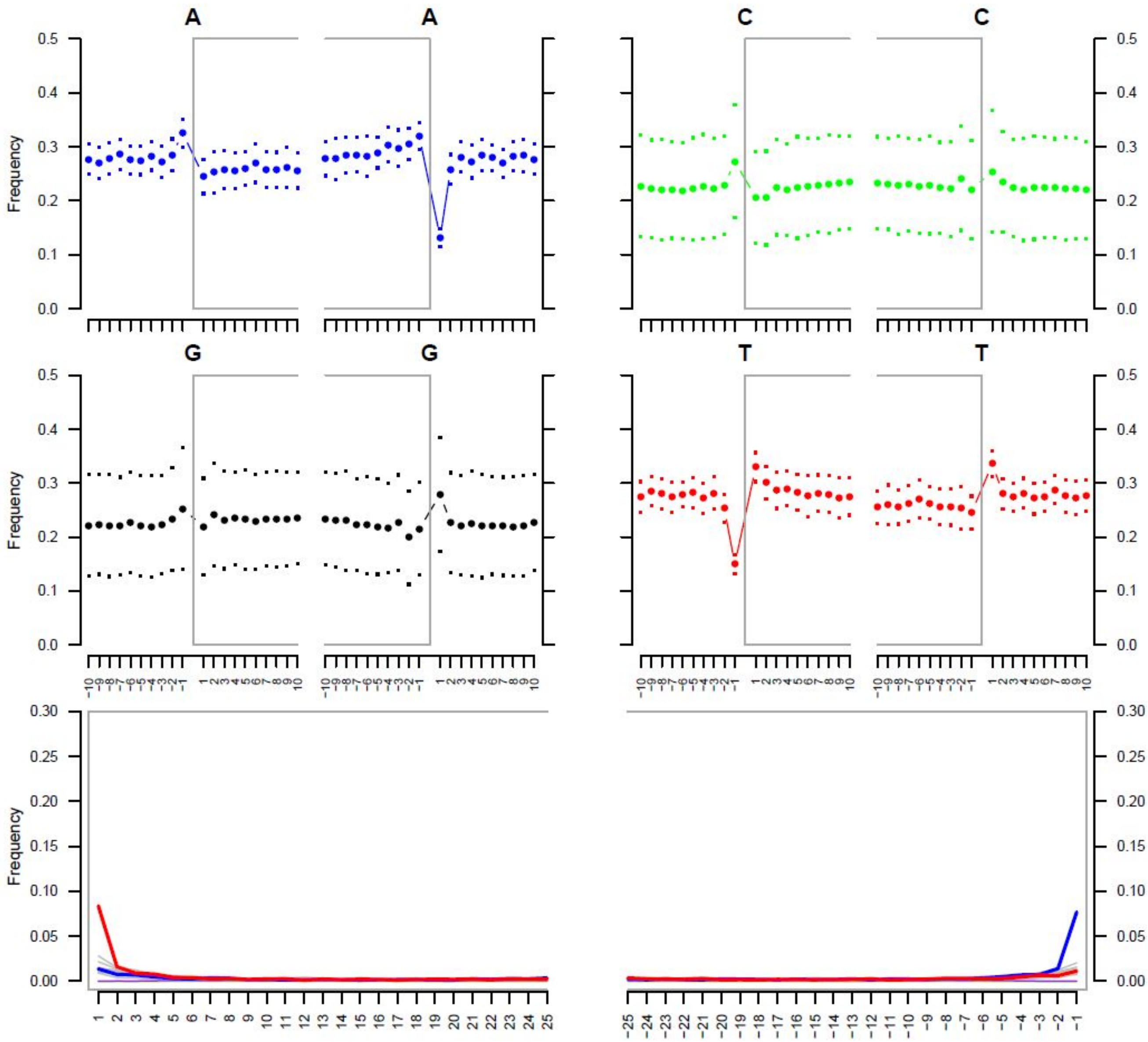

C: CAP1-LIB2. mtDNA capture data for the Harvard Library (83,972 reads). The deamination signal is higher at the terminal ends and absent in the following bases since the library preparation included a UDG treatment that resulted in inefficient removal of 5’ Uracils (the enzyme needs to flip the strand to remove uracil and cannot do it at terminal positions).Efficient Uracil removal occurred starting at base 2.

Figure S3. DNA length distribution in shotgun sequencing data. A. Lib1. The length distribution is very similar to the one produced with only mtDNA reads and the mode of the curve is still around 47 bp. B. Lib2. The DNA length is, on average smaller than with Lib1. Most reads range between 25 bp and 70 bp.

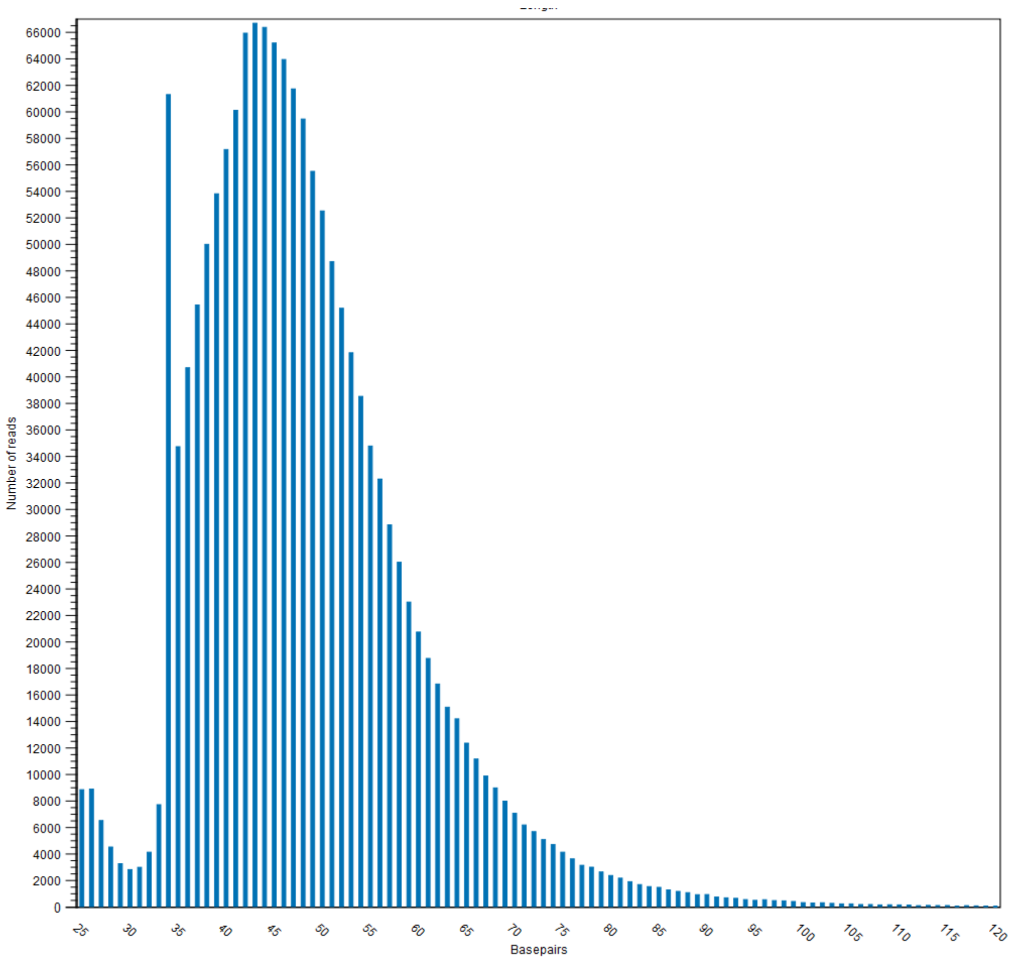

A. A. Lib1- 1,595,239 reads

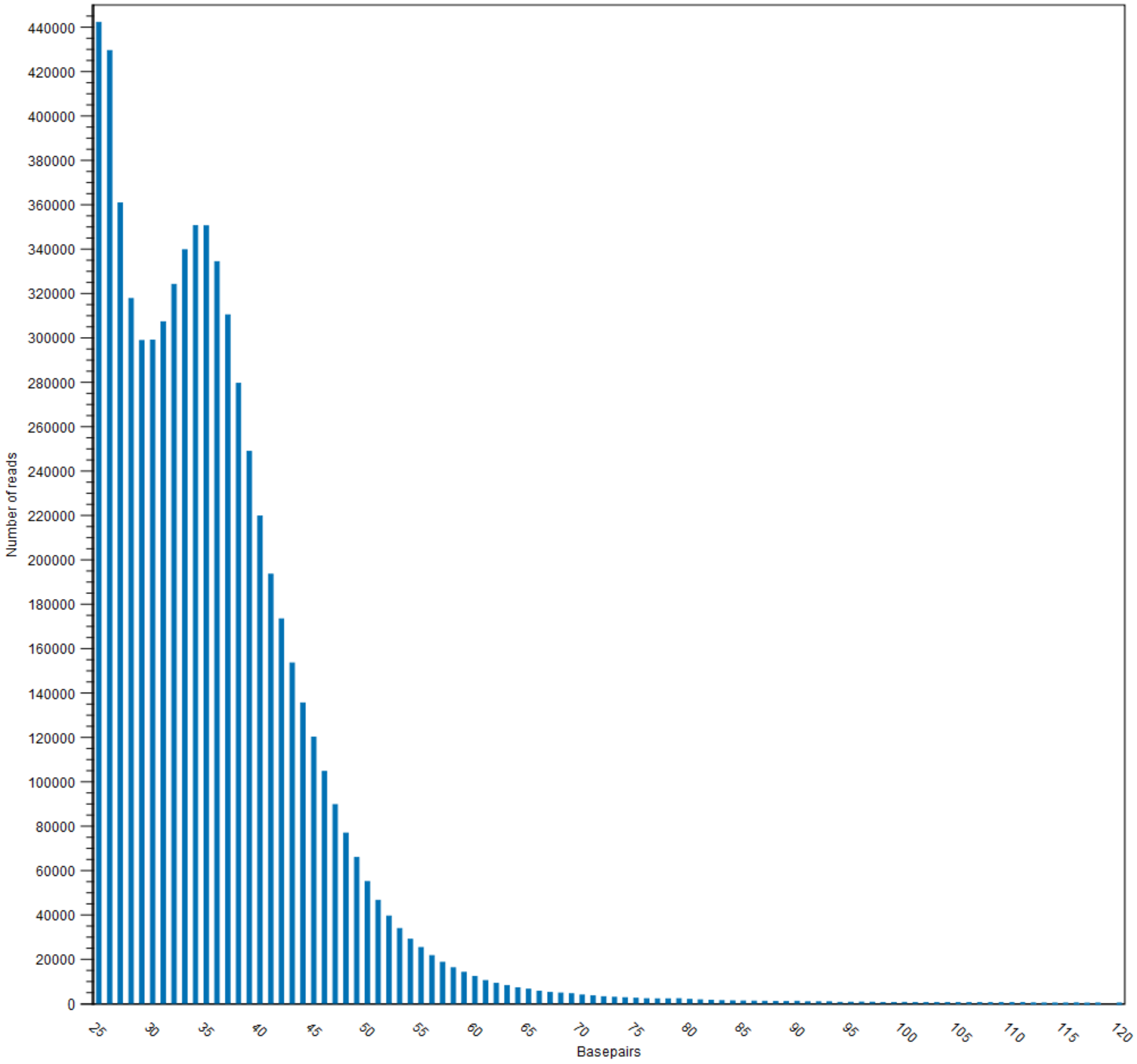

B. B. Lib2- 7,691,326 reads

Reference

Briggs, A. W.; Stenzel, U.; Johnson, P. L.; Green, R. E.; Kelso, J.; Prufer, K.; Meyer, M.; Krause, J.; Ronan, M. T.; Lachmann, M.; al., e., Patterns of damage in genomic DNA sequences from a Neandertal. *Proc Natl Acad Sci U S A* **2007,** *104*, 14616-14621.
