# Supplementary material for: Biological Sexing of a 4000-Year-Old Egyptian Mummy Head to Assess the Potential of Nuclear DNA Recovery from the Most Damaged and Limited Forensic Specimens
Source: Genes (Basel). 2018 Mar 1;9(3):135. doi: 10.3390/genes9030135 (PMC5867856; doi:10.3390/genes9030135)
Supplement: Supplementary file 1 [file genes-09-00135-s001.zip › Sup files/Supplementary Material.pdf]

Supplementary Material

Sample preparation at the FBI Laboratory

Following extraction of the tooth from the jaw, it was sent to an ancient DNA Laboratory where it was gently washed with a sterile wipe soaked with 10% bleach, rinsed with DNA free water and UV irradiated for 10 minutes on each side. The tooth was then embedded in epoxy and divided in two parts. The half containing the crown was sent to the FBI Laboratory and all manipulations were performed in a room dedicated to ancient DNA work. The internal flat surface of the epoxy was gently cleaned with ethanol, sanded to remove potential contaminants and cleaned again with ethanol. The tooth was then slowly drilled and 105 mg of powder were recovered and incubated overnight at 55 °C in 3.5ml of EDTA 0.5M and 100μl of proteinase K (20mg/ml). An extraction reagent blank (RB) was processed alongside the sample to monitor contamination of chemicals. The following day, lysates were transferred to Amicon® Ultra-4 Centrifugal Filter Units (30kDa; Millipore) and centrifuged until the solution levels ranged between 100 and 120μl. The extract and RB were subsequently purified with the MinElute PCR purification kit (Qiagen) following the manufacturer's instructions, and eluted in a low binding tube with 100μl DNA of Elution buffer (EB: 10mM Tris.HCl, pH 8.5).

Both the extract and RB were treated with 3μl of the USER™ Enzyme kit (New England BioLabs; NEB) for one hour and were subsequently purified with the MinElute PCR Purification kit. DNA was eluted in 60μl of EB buffer and quantified using the Qubit 2.0 Fluorometer and the High Sensitivity DNA Assay Kit (Invitrogen). The concentration of dsDNA in the extract was 100pg/μl. No quantification could be obtained with the RB.

50μl of extract or RB were converted into Illumina libraries (Lib1, Lib1/RB); a third library was initiated at this stage with 50μl of DNA-free water (Lib1/NC). The following two kits were used for library preparation: NEBNext Ultra II DNA Library Prep kit for Illumina and the NEBNext Multiplex oligos for Illumina kit that contains looped adapters.

Libraries were amplified for 19 cycles with the NEBNext Ultra II Q5 PCR reagents provided in the NEBNext Ultra II DNA Library Prep kit and dual indexed primers from the NEBNext Multiplex oligos for Illumina kit. After PCR product purification with 1.3x AmPure XP beads (Beckman Coulter), DNA was eluted in 25μl EB and dsDNA was quantified using the Qubit Fluorometer and the dsDNA Broad Range Assay kit. The dsDNA concentration of Lib1 was 83.85 ng/μl.

Sample preparation at Harvard Medical School (HMS)

In a dedicated ancient DNA clean room at Harvard Medical School, 68 mg powder were obtained from the same part of the tooth used for Lib1 by drilling and DNA was extracted following established protocols that retain short DNA molecules [1,2] with a modification that uses manufactured spin column-funnel assemblies from the Roche High Pure Viral Nucleic Acid Large Volume Kit. DNA was eluted in 90μl of TET buffer (Tris EDTA + 0.05% Tween20). An extraction negative control (RB) with no bone powder was also carried through the extraction process to monitor contamination of chemicals.

10μl of extract, RB, and a NC using water instead of extract were used in a 52.2μl reaction containing 3.6U USER^{TM} (NEB), 6μl 10x buffer Tango, 0.24μl 25 mM dNTP mix, 0.6μl 100Mm ATP and incubated at 37°C for 30 minutes. UDG was then inactivated by adding 7.2U Uracil Glycosylase Inhibitor (UGI, NEB) and incubation for 30 min at 37°C. Finally, 4μl T4 polynucleotide kinase (Thermo Fisher) and 1.2μl T4 DNA polymerase (Thermo Fisher) were added, and the final blunt end repair reactions (60μl) were incubated for 15 min at 25°C, followed by 5 min incubation at 12°C. Libraries were then purified using the MinElute PCR purification kit and DNA eluted in 18 μl EB.

Partial double-stranded adapters (for both sides) were prepared by hybridizing one long oligonucleotide containing an Illumina-specific universal adapter sites and a 7bp barcode to a short Illumina-specific universal adapter containing the 7bp reverse complementary barcode. Barcoded adapters were ligated to the blunt end repaired DNA using 4μl T4 DNA ligase buffer, 4μl 50% PEG-4000, 1μl T4 DNA ligase (Thermo Fisher) during a 30 min incubation at room temperature in a final volume of 40μl. Libraries were then purified using the MinElute PCR purification kit and DNA eluted in 16 μl EB. The short part of the barcoded adapter was replaced during a fill-in step by using 16U Bst Polymerase 2.0 (NEB) with 0.25μl 25mM dNTPs, 2.5ul 10x Isothermal amplification buffer in a final volume of 25μl and incubated for 20min at 37°C followed by a heat inactivation at 80°C for 20min. The entire 25 μl of the fill-in step were amplified for 30 cycles with 5U Pfu Turbo Cx Hotstart DNA Polymerase (Agilent), 10μl 10x Pfu Turbo Cx Reaction buffer, 1ul 25mM dNTPs and 10μl of 10μM each primer (PreHybF, PreHybR) and PCR products were purified with the MinElute PCR purification kit and DNA eluted in 50 μl TE buffer with 0.05% Tween. Libraries were quantified using a Nanodrop 8000. The concentration of the library (Lib2) made with the DNA extract was 85 ng/μl. Aliquots of Lib2, Lib2/RB and Lib2/NC were sent to the FBI Laboratory.

Hybridization capture and sequencing at the FBI

In solution hybridization captures were performed on both Lib1 and Lib2 at the FBI laboratory, using a MyBait1 kit from Arbor Biosciences (Ann Arbor, MI) that contains 100 ng of 80 bp RNA biotinylated baits designed by the company to capture the human mitochondrial DNA Genome (mtGenome). All 14,801 baits were designed with at least 5x tiling (16 bp probe spacing) and

were complementary to the sequences of 197 human mitochondrial sequences. Capture was performed according to the manufacturer's protocol (Version 3.01 August 2015), with the exception of the hybridization temperature, which was lowered to 62°C. After capture, DNA was amplified with the Kapa Hifi Hotstart Ready mix (Kapa Biosystems/Roche) for 15 cycles. PCR products were purified and quantified on a Bioanalyzer. Post captured indexed libraries were pooled and sequenced as paired end reads first at the FBI Laboratory on an Illumina MiSeq Desktop Sequencer with a MiSeq Reagent Kit v2 (2x150, + 2x8 cycles) and then twice at NBFAC on a HiSeq 2500 with 2x250 cycles + 2x8 cycles.

Hybridization capture and sequencing at the HMS

Another enrichment was performed on Lib2 in the HMS Laboratory. A hybridization capture enrichment following [3] with the baits replaced by an oligo pool (synthesized on a microarray by CustomArray Inc. that were designed using a 3bp tiling of the RSRS reference genome [4] and nuclear SNPs spiked in (unpublished). Dual indices were then added to the enriched library during the final amplification step and the product was sequenced together with other libraries on a NextSeq500 with a 150cycles v2 High Output kit for 2x76 cycles+ 2x7 cycles.

R_X and R_Y calculations

1. **R_X**

$$R_x = \frac{\sum_{i=1}^{22} \frac{\rho_x}{\rho_i}}{22}$$

Where *f1*, *f2*,. . ., *f22*, and *fx* denote the number of sequences aligning to each chromosome relative to the total number of sequences aligning to autosomes and sex chromosomes. {*fi*}(i = 1, 2,. . ., 22, x) is estimated directly from the number of sequences aligning to all autosomes. The 95% CI was again computed as Rx ± 1.96 SE where SE is the standard error measuring the amount of variability in the *Rx* mean compared to the 22 autosomes. The 95% CI was computed as *Rx*±1.96SE. SE is the standard error measuring the amount of variability in the *Rx* mean compared with 22 autosomes [5].

2. **R_Y**

RY= number of sequences from the Y chromosome (n_Y)/ number of sequences from the X and Y chromosomes ($n_Y + n_X$). The 95% confidence interval (CI) was computed as $R_Y \pm 1.96$ Standard error (SE) where SE= $\surd R_Y \times (1-R_Y)/n_X+n_Y$ [6].

1. Dabney, J.; Knapp, M.; Glocke, I.; Gansauge, M. T.; Weihmann, A.; Nickel, B.; Valdiosera, C.; Garcia, N.; Paabo, S.; Arsuaga, J. L.; et al. Complete mitochondrial genome sequence of a Middle Pleistocene cave bear reconstructed from ultrashort DNA fragments. *Proc Natl Acad Sci U S A* **2013,** *110*, 15758-15763.
2. Korlevic, P.; Gerber, T.; Gansauge, M. T.; Hajdinjak, M.; Nagel, S.; Aximu-Petri, A.; Meyer, M., Reducing microbial and human contamination in DNA extractions from ancient bones and teeth. *BioTechniques* **2015,** *59*, 87-93.
3. Maricic, T.; Whitten, M.; Paabo, S., Multiplexed DNA sequence capture of mitochondrial genomes using PCR products. *PloS one* **2010,** *5*, e14004.
4. Behar, D. M.; van Oven, M.; Rosset, S.; Metspalu, M.; Loogvali, E. L.; Silva, N. M.; Kivisild, T.; Torroni, A.; Villems, R., A "Copernican" reassessment of the human mitochondrial DNA tree from its root. *Am. J. Hum. Genet.* **2012,** *90*, 675-684.
5. Mittnik, A.; Wang, C. C.; Svoboda, J.; Krause, J., A Molecular Approach to the Sexing of the Triple Burial at the Upper Paleolithic Site of Dolni Vestonice. *PloS one* **2016,** *11*, e0163019.
6. Skoglund, P.; Storå, J.; Götherström, A.; Jakobsson, M., Accurate sex identification of ancient human remains using DNA shotgun sequencing. *J. Archaeol. Sci.* **2013,** *40*, 4477-4482.
